# Permanent Peripheral Neuropathy: A Case Report on a Rare but Serious Debilitating Side-Effect of Fluoroquinolone Administration

**DOI:** 10.1177/2324709614545225

**Published:** 2014-07-27

**Authors:** Jacquelyn K. Francis, Elizabeth Higgins

**Affiliations:** 1 Albany Medical College, Albany, NY, USA

**Keywords:** fluoroquinolones/adverse effects, drug labeling, peripheral nerve injuries/chemically induced, Food and Drug Administration, peripheral nervous system disease

## Abstract

The health risks and side effects of fluoroquinolone use include the risk of tendon rupture and myasthenia gravis exacerbation, and on August 15, 2013, the Food and Drug Administration updated its warning to include the risk of permanent peripheral neuropathy. We present a case of fluoroquinolone-induced peripheral neuropathy in a patient treated for clinically diagnosed urinary tract infection with ciprofloxacin antibiotic.

## Introduction

While there has been success in recent years in decreasing the numbers of unnecessary antibiotic administrations,^1^ still rampant in medical practice is the inappropriate use of antibiotics. Fluoroquinolones administration is no different. These bactericidal agents are capable of central nervous system (CNS) penetration,^2^ with an impressive treatment profile that includes an enhanced spectrum of activity, high oral bioavailability, high serum drug concentration that parallels that of intravenous drug administration, and rapid mechanism of action. It is for this reason that physicians favor these drugs for treatment of simple infections, which range from uncomplicated urinary tract infections (UTIs) and gastrointestinal infections to lower respiratory infections and pneumonias. According to established guidelines, however, these antibiotics are recommended as drugs of last resort and for treatment of cases refractory to other safer antibiotic alternatives. Reports in recent years of the adverse drug events of these drugs are on the rise, with not only an overrepresentation of common antibiotic complaints, including diarrhea, nausea, and headache that occur at rates higher than most other antimicrobials on the market,^3^ but there is also mounting evidence suggesting the potential for long-term adverse peripheral nervous system (PNS) effects from fluoroquinolone usage. The need for physicians to be judicious when prescribing these drugs is therefore paramount.

## Case Presentation

A 57-year-old Caucasian female presented to outpatient clinic with complaints of dysuria, polyuria, and urinary urgency. Urinalysis showed 2+ leukocytes and trace blood. Based on her clinical presentation, she was treated for UTI with a ciprofloxacin regimen of 250 mg twice a day for 5 days. Subsequent urine culture showed no evidence of organism, and against advice for reevaluation, she was lost to follow-up. She presented 2 months later reporting whole body burning and alopecia. The burning, she claimed, started 2 or 3 days after completion of the prescribed course of ciprofloxacin. The burning lasted 3 weeks and resolved only to recur, unrelentingly, 3 weeks later. She had been unable to adorn clothing during this time, for she said this triggered whole body burning. At the point wherein she was finally able to wear clothing, she presented to the clinic. Hydration and Epsom salt soaks provided no relief. She reported pain of 10/10. Her past medical history is significant for trigeminal neuralgia, in remission for 12 years. The patient was on no medications at the time of her visit. She has no specific medication allergies, but does get gastrointestinal symptoms with opioids, namely, fentanyl. Physical examination was unremarkable. Vitals at the time that she was seen included the following: blood pressure 132/78 mm Hg, temperature of 97°F, heart rate of 60 beats per minute, respirations of 18. Her body mass index was 17.94, down from 20.3 two months earlier. On detailed neurologic examination, cranial nerves II through XII were intact bilaterally. There was no pronator drift of outstretched arms. There was some muscle wasting in biceps; however, overall tone was normal. Strength was full bilaterally. Reflexes were 2+ and symmetric at the biceps, triceps, knees, and ankles. Plantar responses were flexor. Light touch and pinprick produced pain and paresthesias diffusely in the upper and lower extremities; however, position sense and vibration sense were intact in fingers and toes. Rapid alternating movements and fine finger movements were intact. There was no dysmetria on finger-to-nose and heel-knee-shin. There were no abnormal or extraneous movements. Romberg was absent. The patient’s posture was normal. Gait was steady with normal, though tentative, steps, base, arm swing, and turning. Heel and toe walking were normal. Tandem gait was normal. She had no discernable rash or skin lesions.

Subsequent complete blood work analysis to check for an electrolyte abnormality basis of her complaints was unremarkable. Her complete blood count was normal with a hematocrit of 41%. Her vitamin B_12_ level was 258 pg/mL, with a normal range of 200 to 900 pg/mL. Her thyroid stimulating hormone level was 2.05, with a normal range of 0.4 to 6.0. Her immunoglobulin levels were normal. Her vitamin D level was 13 nmol/L (optimal >30 nmol/L). Copper level was 98 mg (normal 50-80 mg). Vitamin E was normal at 12.7 µg/mL (normal range = 5.5-17 µg/mL). Vitamin B_1_ was normal at 5.4 µg/dL (normal range = 2.5-7.5 µg/dL).

Her blood work and further questioning could provide no new medical etiology for her symptoms, and so the patient was subsequently sent for complete neurological workup. Workup included heavy metal toxicity screening to assess for possible heavy metal exposure to lead, mercury, cadmium, and zinc. Electrophysiological studies were also done to assess neuromuscular nerve action potential transmission, a test that could discern a neuromuscular disorder etiology. Three-millimeter skin punch biopsy to assess for small fiber density and possible neurologic process were also done. These tests were all negative. Neurological workup could not determine a unique cause of her symptoms. It was concluded that if her symptoms were neurologic-based, it was, in fact, a multifocal process.

Two years after the initial onset of symptoms, the patient continues to suffer from polyneuropathies chronologically related to ciprofloxacin use. At her most recent visit, she describes constant pain of 7/10 and is unable, she states, to ambulate for more than 2 minutes, without intense shooting pains up and down her lower extremities. She describes “pins and needles” up and down her legs and thighs radiating to her buttocks and feet. She claims that her upper body and abdomen have now been spared of such feelings. She describes severe alopecia and ambulates now with a broad-based gait. She describes being on permanent disability because of her condition. The rest of her physical examination remains unchanged. There are no gross neurological deficits discernible on neurologic examination. The patient remains on amitriptyline 20 mg daily for control of her pain symptoms.

## Discussion

Fluoroquinolones are fluorinated quinolones, the only bactericidal agent in the antibiotic class capable of directly inhibiting DNA synthesis. They do this by promoting cleavage of bacterial DNA in the DNA–enzyme complexes of DNA gyrase and topoisomerase IV.^2^ Generally, gram-negative antibacterial activity correlates with inhibition of DNA gyrase, and gram-positive antibacterial activity corresponds with inhibition of DNA type IV topoisomerase.^2,4^ With the introduction of these drugs in the 1960s, physicians were able, for the first time, to treat severe gram-negative infections orally.^3^ The first successful fluorination of part of the quinolone drug in 1986, in the form of norfloxacin, brought with it the capability of crossing the blood–brain barrier and achieving CNS penetration.^5^ This and the already great treatment profile in the form of enhanced spectrum of activity, high oral bioavailability, high serum drug concentration comparable to intravenous infusion, and rapid mechanism of action added to the popularity of these drugs ultimately resultingin the indiscriminate use of these drugs. The enhanced treatment profile of these drugs came at a price however, with adverse effects so severe that use of many fluoroquinolones since then being restricted or the drugs withdrawn from the market entirely.^6,7^

One of the challenges of diagnosing a patient with fluoroquinolone-associated peripheral neuropathy is the diffuse, confusing, and delayed array of symptoms that can occur. A 1996 study first brought these adverse effects to light.^7^ While patients on the fluorinated drugs exhibited less side effects than those associated with first-generation quinolone predecessors, such as nausea and gastrointestinal disturbances, 0.9% to 1.6% experienced adverse reactions relating to the peripheral and central nervous system, including headache, dizziness, drowsiness, agitation, psychosis, and convulsions, as well as peripheral sensory disturbances, symptoms that had never been complained of prior, at least not on any significant scale. Of these patients, 81% had symptoms occurring within 1 week of drug administration, with paresthesia being the mainly reported symptom. Five years later, a 2001 study found that contrary to previous reports suggesting that fluoroquinolone-associated PNS events are mild and short term, 80% of study participants reported severe events that typically involved multiple organ systems, especially the PNS, with symptom onset as early as 24 hours within initiation of treatment. 58% of these cases had symptoms lasting greater than 1 year.^8^

Another 2001 formal study that sought to assess the prevalence of fluoroquinolone-induced PNS adverse side effects highlighted the severity of these effects. The study concluded that there was a high association between fluoroquinolone antibiotics and severe, long-term adverse PNS and multiple organ system effects that included PNS sensory symptoms (91%), peripheral neuropathy motor symptoms (55%), and CNS effects (75%). Over 80% of the patients surveyed had sequalae stemming from fluoroquinolone use that lasted for greater than 1 year.^9^ A subset of these patients and their adverse drug events are included in Table 1.

**Table 1.** 15 of the 45 Total Reported Cases of Fluoroquinolone-Associated Events in the Literature^6^.

Patient Age/Gender	Drug	Use	Onset of Adverse Symptoms (Days)	Symptoms
32/female	Ciprofloxacin	UTI	1	Tingling and anxiety
11/female	Levofloxacin	Osteomyelitis	5	Tingling “electric” pain in arms and legs
39/male	Levofloxacin	Prostatitis	20	Diffuse tingling, “skin-crawling” sensation, numbness
26/male	Levofloxacin	Bronchitis	1	Numbness, tingling, twitching, allodynia, anxiety
40/male	Levofloxacin	UTI	4	Tingling, burning pain, twitching, joint/muscle pain
41/female	Levofloxacin	Sinusitis	2	Diffuse numbness, allodynia, severe muscle/joint pain
32/male	Levofloxacin	Prostatitis	1	Numbness, tingling, shooting pain
34/male	Levofloxacin	Prostatitis	5	Numbness, twitching, increased sensitivity to temperatures, joint/muscle pain, anxiety
31/female	Levofloxacin	Sinusitis	3	Tingling, burning pain, numbness, “pins and needles,” temperature intolerance
53/male	Levofloxacin	Prostatitis	2	Numbness, tingling, cramps, tremors, joint/tendon pain
49/female	Ofloxacin	Pelvic infection	1	Diffuse numbness, “pins and needles,” burning pain, joint pain, panic attacks
51/female	Levofloxacin	Pelvic infection	2	“Electric” sensations, numbness, tingling, allodynia
56/female	Ofloxacin, ciprofloxacin	UTI	3	Acute nocturnal onset of severe burning pain, numbness, twitching, “electric” sensations, 30-lb weight loss, muscle/joint pain
44/female	Ciprofloxacin	Bronchitis	4	Numbness, allodynia, hyperesthesia, tremor, “electric” sensation, diffuse burning sensation, tremors, twitching, temperature intolerance
38/male	Lomefloxacin	Prostatitis	14	Severe twitching, numbness, “electric” sensations, tingling, muscle/joint pain, hyperesthesia

Abbreviation: UTI, urinary tract infection.

Despite these seemingly significant numbers and overwhelming reports from patients, physicians continue to prescribe fluoroquinolone antibiotics unsystematically, against US Food and Drug Administration recommendations. The pressures of health care facilities and patients alike to increase patient turnaround and quickly alleviate symptoms may compound this problem ^10^.

As highlighted in the aforementioned case, the peripheral neuropathy reported with fluoroquinolone administration can be severe, debilitating, and permanent. It is for this reason that physicians need to practice due diligence when prescribing not only antibiotics, but any drug. Physicians also need to practice vigilance in the event of an adverse reaction. They can do this with careful follow-up of patients and ensure that patients are aware of all the side effects that may be associated with their prescribed drug. Patients need to know what to look for and where to go in the event that one of these symptoms become manifest. It is our hope that the updated FDA warning and presentation of this case will encourage physicians to be more conscientious of their treatment selections.

## Take Home Points

- The FDA recommends that fluoroquinolones be used as a drug of last resort and for treatment of cases refractory to other safer antibiotic alternatives.
- The FDA updated their black box warnings on all fluoroquinolones to stress the rapidity of onset and permanence of peripheral neuropathy associated with their use.
- Physicians should be aware of the risks and side effects associated with the drugs that are prescribed and be able to inform patients of the risks associated with the use of these drugs.
- Physicians should always aim to administer the least broad spectrum antibiotic possible based on known sensitivities and regional resistance patterns.
